# Supplementary material for: Neuropeptide ACP facilitates lipid oxidation and utilization during long-term flight in locusts
Source: eLife. 2021 Jun 21;10:e65279. doi: 10.7554/eLife.65279 (PMC8324298; doi:10.7554/eLife.65279)
Supplement: Supplementary file 2. [file elife-65279-supp2.docx]

Supplementary File 2. Mutation efficiency of G0 and G1 generation of ACP mutants.

| Generations | Embryos | Survival rate | Mutant efficiency |
| --- | --- | --- | --- |
| G0 | 162 (injected) | 52 (31.52%) | 44 (84.62%) |
| G1 | 201 | 155 (77.11%) | 74 (47.74%) |
