# Supplementary material for: Neuropeptide ACP facilitates lipid oxidation and utilization during long-term flight in locusts
Source: eLife. 2021 Jun 21;10:e65279. doi: 10.7554/eLife.65279 (PMC8324298; doi:10.7554/eLife.65279)
Supplement: Supplementary file 3. [file elife-65279-supp3.docx]

Supplementary File 3. Mutation efficiency of G0 and G1 generation of ACPR mutants.

| Generations | Embryos | Survival rate | Mutant efficiency |
| --- | --- | --- | --- |
| G0 | 288 (injected) | 108 (37.5%) | 56 (51.85%) |
| G1 | 188 | 136 (72.34%) | 62 (45.59%) |
