# Supplementary material for: Neuropeptide ACP facilitates lipid oxidation and utilization during long-term flight in locusts
Source: eLife. 2021 Jun 21;10:e65279. doi: 10.7554/eLife.65279 (PMC8324298; doi:10.7554/eLife.65279)
Supplement: Supplementary file 4. [file elife-65279-supp4.docx]

Supplementary File 4. Primers for qPCR, gRNA synthesis, and RNAi experiment.

| **Genes** | **Forward primer**  **(5’-3’)** | **Reverse primer**  **(5’-3’)** |
| --- | --- | --- |
| **ACP qPCR** | GCAAGGCTGCTCCTCACA | GTTGGCGAAGTTCGTTCA |
| **AKH2 qPCR** | TAGTGGTGGCGATGTTGG | TGTATGAGCCTGTAGAGGAACG |
| **NPF1 qPCR** | AACCTCAGAGTCTAAAAATA | GTAACAGTCGTGAAACCACA |
| **GPB5 qPCR** | CTGCTGCTGCTGTCGGTGGT | GCGTCGGTCTTGGTGACCTTGT |
| **AKH3 qPCR** | GCGCAGCTCAACTTCACGC | CGCCTCTGATGTAAACCTGCTG |
| **AST-A qPCR** | CAGTGATGCCGCAGGAGGT | CTCGGACACGTAGGTGTAGGC |
| **CAPA qPCR** | ATGGCCGCCCCCAGCACCAG | GCTGCTGCTCTGAAACGTTG |
| **DH46 qPCR** | GCAGACGCCCAGTTTCAGG | AGCGCTTGCCGATCTGCTG |
| **ILP qPCR** | ATGATGTGGAAGCTGTGCCT | CTCCGAGTCTGACACATCT |
| **OMP qPCR** | ATGTCCCCGGTGCGAGTGC | CTGCAGCAGCAGGCGCCG |
| **ACPR qPCR** | CACCCCAAGGACCCCAACT | AGTACATGGCGATCACGCAG |
| **FABP qPCR** | GCCGCAAGGTCAAGTCTATC | TATTCTCGTCGCCACCAAGT |
| **CROT qPCR** | CGGCTTCGGCAAGGAGTT | CGCACCCACTCCATCTGTTC |
| **CPT2 qPCR** | ACTTGGGTTCCAGGTTGC | CTGTCAAATCCCTGTCCC |
| **ACDM qPCR** | TCATTGTGGTGGACTTGG | TCTTCAATCAGGCGTCCC |
| **ACADS qPCR** | TGCTTTGAGTGAACCAGGAA | ACTAACAAGGAAGGCACT |
| **ACADSB qPCR** | CTGCCTTGGTTGACATAC | CGATTGTGGCTCCGATAAAC |
| **ECH-6 qPCR** | ACTTCTTGGGTCACTGGA | TGACTTCCCAACCATTCG |
| **ACAT1-qPCR** | TTTTCGTGGGAGTATGGC | ATTCCTGAGGCACAAACC |
| **CRAT qPCR** | GGCTTCACGCTTCCTACT | TCCAGGGATTCGGCAGGT |
| **rp49 qPCR** | CGCTACAAGAAGCTTAAGAGGTCAT | CCTACGGCGCACTCTGTTG |
| **ACP_genome_** | GCACTCTGGGGGATGTAACT | CAAGGGAGGAATCGTAGCAT |
| **ACPR_genome_** | CGCACTTTCAAGGCTAA | CTGTAGGGTGTCCATTATTT |
| **ACP RNAi** | TAATACGACTCACTATAGG  AGATACAAGCAGGGTGAA | TAATACGACTCACTATAGG  CTTCTGTTGGGTACTTCTA |
| **AKH2 RNAi** | TAATACGACTCACTATAGG  GGCGACCACAAACAGGATG | TAATACGACTCACTATAGG  GCTTCCTCGCCTCAATCTGT |
| **NPF1 RNAi** | TAATACGACTCACTATAGG  GCTACTACTCGCAGGTGG | TAATACGACTCACTATAGG  CGGATGTCGTCGATGACG |
| **GPB5 RNAi** | TAATACGACTCACTATAGG  CTGCTGCTGCTGTCGGTGGT | TAATACGACTCACTATAGG  GCGTCGGTCTTGGTGACCTTGT |
| **FABP RNAi** | TAATACGACTCACTATAGG  GGTCCACGAGCAGAAGG | TAATACGACTCACTATAGG  CACACCAAGCTCATATTAC |
| **GFP RNAi** | TAATACGACTCACTATAGG  CACAAGTTCAGCGTGTCCG | TAATACGACTCACTATAGG  GTTCACCTTGATGCCGTTC |
| **ACPgRNA** | TAATACGACTCACTATAGG  CATGGTGCTGCTACCTGG | TTCTAGCTCTAAAACCCA  GGTAGCAGCACCATGCC |
| **ACPRgRNA** | TAATACGACTCACTATAGG  TGCAGCACGGCGAAGTAG | TTCTAGCTCTAAAACCTA  CTTCGCCGTGCTGCACC |

Red font indicates T7 promoter sequence.
