# Supplementary material for: Neuropeptide ACP facilitates lipid oxidation and utilization during long-term flight in locusts
Source: eLife. 2021 Jun 21;10:e65279. doi: 10.7554/eLife.65279 (PMC8324298; doi:10.7554/eLife.65279)
Supplement: Transparent reporting form [file elife-65279-transrepform.pdf]

eLIFE
1st Floor
24 Hills Road
Cambridge CB2 1JP, UK
P 01223 855340
W elifesciences.org
T @elife

# *eLife's* transparent reporting form

We encourage authors to provide detailed information *within their submission* to facilitate the interpretation and replication of experiments. Authors can upload supporting documentation to indicate the use of appropriate reporting guidelines for health-related research (see EQUATOR Network), life science research (see the BioSharing Information Resource), or the ARRIVE guidelines for reporting work involving animal research. Where applicable, authors should refer to any relevant reporting standards documents in this form.

If you have any questions, please consult our Journal Policies and/or contact us: editorial@elifesciences.org.

**Sample-size estimation**

- You should state whether an appropriate sample size was computed when the study was being designed
- You should state the statistical method of sample size computation and any required assumptions
- If no explicit power analysis was used, you should describe how you decided what sample (replicate) size (number) to use

Please outline where this information can be found within the submission (e.g., sections or figure legends), or explain why this information doesn't apply to your submission:

The sample size is determined according to the previous studies in two locust species, *Locusta migratoria* and *Schistocerca gregaria* (Yang et al., 2014, PLoS Genetics, 10:e1004206; Hou et al., eLife, 6:e22526; Rajapakse et al., 2019, Journal of Experimental Biology, 222:jeb203455).
For the analysis of gene expression, each sample is composed of 3-10 individuals depending on tissues detected.
For the measurement of metabolites, each sample is composed of 3-4 individuals.
For flight experiments, each sample composed more than 20 individuals according to previous study (Rajapakse et al., 2019, Journal of Experimental Biology, 222:jeb203455).
Detailed information of sample size and biological replicate number for each experiment can be found in the figure legends.

**Replicates**

- You should report how often each experiment was performed
- You should include a definition of biological versus technical replication
- The data obtained should be provided and sufficient information should be provided to indicate the number of independent biological and/or technical replicates
- If you encountered any outliers, you should describe how these were handled
- Criteria for exclusion/inclusion of data should be clearly stated
- High-throughput sequence data should be uploaded before submission, with a private link for reviewers provided (these are available from both GEO and ArrayExpress)

Please outline where this information can be found within the submission (e.g., sections or figure legends), or explain why this information doesn't apply to your submission:

eLife Sciences Publications, Ltd is a limited liability non-profit non-stock corporation incorporated in the State of Delaware, USA, with company number 5030732, and is registered in the UK with company number FC030576 and branch number BR015634 at the address 1st Floor, 24 Hills Road, Cambridge CB2 1JP | August 2014

- In this work, all experiments were carried out for at least 3 independent biological replicates.
- The number of biological replications for each experiment is shown in the corresponding figure legend.
- We exclude the data that do not meet normal distribution for the analysis of gene expression, biochemistry assay, and flight activity. For flight activity analysis, locusts that do not flight were excluded (Method Parts-Statistical analysis).
- The data of peptidome and metabolic have been uploaded as supplementary data and source data, and the complete RNA-seq data have been deposited in the Genome Sequence Archive (Genomics, Proteomics & Bioinformatics 2017) in National Genomics Data Center, Beijing Institute of Genomics (China National Center for Bioinformation), Chinese Academy of Sciences, under accession number CRA003348 that are publicly accessible at https://bigd.big.ac.cn/gsa (Method Parts-RNA-seq and analysis).

**Statistical reporting**

- Statistical analysis methods should be described and justified
- Raw data should be presented in figures whenever informative to do so (typically when N per group is less than 10)
- For each experiment, you should identify the statistical tests used, exact values of N, definitions of center, methods of multiple test correction, and dispersion and precision measures (e.g., mean, median, SD, SEM, confidence intervals; and, for the major substantive results, a measure of effect size (e.g., Pearson's r, Cohen's d)
- Report exact p-values wherever possible alongside the summary statistics and 95% confidence intervals. These should be reported for all key questions and not only when the p-value is less than 0.05.

Please outline where this information can be found within the submission (e.g., sections or figure legends), or explain why this information doesn't apply to your submission:

We have described the statistical analysis methods in detail in the Materials and Methods part.

We have provided the raw data for figures with N < 10 as source data.

We have identified the statistical tests for each experiment. Detailed information is labeled in figure legends. In addition, exact p-values for are also shown in the figure legend.

(For large datasets, or papers with a very large number of statistical tests, you may upload a single table file with tests, Ns, etc., with reference to sections in the manuscript.)

**Group allocation**

- Indicate how samples were allocated into experimental groups (in the case of clinical studies, please specify allocation to treatment method); if randomization was used, please also state if restricted randomization was applied
- Indicate if masking was used during group allocation, data collection and/or data analysis

Please outline where this information can be found within the submission (e.g., sections or figure legends), or explain why this information doesn't apply to your submission:

The samples were allocated into experimental group randomly, and no restricted randomization was applied (Methods part-Tethered Flight Assay).

No masking was used during group allocation, data collection and data analysis.

**Additional data files ("source data")**

- We encourage you to upload relevant additional data files, such as numerical data that are represented as a graph in a figure, or as a summary table
- Where provided, these should be in the most useful format, and they can be uploaded as "Source data" files linked to a main figure or table
- Include model definition files including the full list of parameters used
- Include code used for data analysis (e.g., R, MatLab)
- Avoid stating that data files are "available upon request"

Please indicate the figures or tables for which source data files have been provided:

Figure 1-source data. Raw data for comparisons of peptide contents, gene expression, and flight activity.
Figure 2-source data. Raw data for gene expression and flight activity.
Figure 3-source data. Raw data for gene expression and flight activity.
Figure 4-source data. Raw data for RNA-seq and gene expression.
Figure 5-source data. Raw data for metabolic and metabolite contents.
Figure 6-source data. Raw data for flight activity, metabolite contents, and gene expression.

eLife Sciences Publications, Ltd is a limited liability non-profit non-stock corporation incorporated in the State of Delaware, USA, with company number 5030732, and is registered in the UK with company number FC030576 and branch number BR015634 at the address 1st Floor, 24 Hills Road, Cambridge CB2 1JP | August 2014
